# Hippo kinases Mst1 and Mst2 maintain NK cell homeostasis by orchestrating metabolic state and transcriptional activity

**DOI:** 10.1038/s41419-024-06828-x

**Published:** 2024-06-19

**Authors:** Peiran Feng, Liang Luo, Quanli Yang, Wanqing Meng, Zerong Guan, Zhizhong Li, Guodong Sun, Zhongjun Dong, Meixiang Yang

**Affiliations:** 1 Guangdong Provincial Key Laboratory of Spine and Spinal Cord Reconstruction, The Fifth Affiliated Hospital of Jinan University (Heyuan Shenhe People’s Hospital), Jinan University, Heyuan, 517000 China; 2 The Biomedical Translational Research Institute, School of Medicine, Jinan University, Guangzhou, 510632 China; 3 Guangdong Provincial Key Laboratory of Tumor Interventional Diagnosis and Treatment, Zhuhai Institute of Translational Medicine, Zhuhai People’s Hospital（Zhuhai Clinical Medical College of Jinan University）, Jinan University, Zhuhai, 519000 China; 4 Department of Orthopedics, The First Affiliated Hospital, Jinan University, Guangzhou, 510630 China; 5 The First Affiliated Hospital of Anhui Medical University and Institute for Clinical Immunology, Anhui Medical University, 230032 Anhui, China; 6 Key Laboratory of Ministry of Education for Viral Pathogenesis & Infection Prevention and Control (Jinan University), Guangzhou Key Laboratory for Germ-free animals and Microbiota Application, Institute of Laboratory Animal Science, School of Medicine, Jinan University, Guangzhou, 510632 China

**Keywords:** Immune cell death, NK cells

## Abstract

Natural killer (NK) cells play a crucial role in immune response against viral infections and tumors. However, further investigation is needed to better understand the key molecules responsible for determining the fate and function of NK cells. In this study, we made an important discovery regarding the involvement of the Hippo kinases Mst1 and Mst2 as novel regulators in maintaining mouse NK cell homeostasis. The presence of high Mst1 and Mst2 (Mst1/2) activity in NK cells is essential for their proper development, survival and function in a canonical Hippo signaling independent mode. Mechanistically, Mst1/2 induce cellular quiescence by regulating the processes of proliferation and mitochondrial metabolism, thereby ensuring the development and survival of NK cells. Furthermore, Mst1/2 effectively sense IL-15 signaling and facilitate the activation of pSTAT3-TCF1, which contributes to NK cell homeostasis. Overall, our investigation highlights the crucial role of Mst1/2 as key regulators in metabolic reprogramming and transcriptional regulation for mouse NK cell survival and function, emphasizing the significance of cellular quiescence during NK cell development and functional maturation.

## Introduction

NK cells are the predominant innate lymphoid cells responsible for mediating antiviral and anti-tumor immunity [1]. By integrating activating and inhibitory signals, NK cells recognize and eliminate abnormal cells through the release of cytotoxic granules while stimulating adaptive immune responses [2]. The abnormal quantity and quality of NK cells are highly correlated with tumorigenesis. Therefore, maintaining NK cell homeostasis is essential for innate defense against “unwanted” cells. NK cells undergo a maturation process from hematopoietic stem cells in the bone marrow (BM) to the periphery [3]. It is noteworthy that mouse NK cell precursors (CD3^–^CD122^+^NK1.1^–^CD11b^–^, NKp) initiate the expression of CD122, the β-chain of the IL-15 receptor, which plays a crucial role in committing to the NK cell lineage [4, 5]. Driven by IL-15 signaling, NKp cells further differentiate into immature (CD3^–^CD122^+^NK1.1^+^CD11b^–^, imNK) and mature NK cells (CD3^–^CD122^+^NK1.1^+^CD11b^+^, mNK) [5]. The maturation process of NK cells is accompanied by differential surface marker expression. Based on the differential expression patterns of CD27 and CD11b, NK cells can be further classified into three main developmental phases: immature NK (CD27 SP, CD27^+^CD11b^-^); mature NK (DP, CD27^+^CD11b^+^) and terminal mature NK (CD11b SP, CD27^-^CD11b^+^) [6]. The development of NK cells is regulated by cytokine receptors and influenced by external signals, wherein interleukin-15 (IL-15) emerges as a pivotal factor in both NK cell lineage commitment and terminal maturation [7, 8] IL-15 stimulation primarily activates the JAK-Stat and PI3K-mTOR pathways. Therefore, individuals lacking JAK and STAT5A/B exhibit impaired NK cell development [9–12]. Additionally, we and other research groups have underscored the significant impact of PI3K signaling on NK cell development and function, highlighting the critical role played by energy metabolism-related signaling molecules in governing NK cell development [13–16]. Nonetheless, whether additional signaling pathways are necessary to prevent excessive activation of NK cells and maintain cellular quiescence during their development are unclear.

The conserved Hippo signaling pathway serves as a crucial metabolic-related regulator of cell proliferation and differentiation [17]. The classical Hippo pathway is primarily activated by upstream signals that stimulate Mst1/2 kinases, which in turn phosphorylate and activate downstream Lats1/2 kinases. These proteins further phosphorylate the transcriptional co-activators YAP/TAZ [18]. Moreover, the Hippo signaling pathway can also exert diverse non-classical regulatory effects through phosphorylation of non-Hippo pathway molecules or through cross-talk with other signaling pathways, such as PI3K-Akt, Wnt and Notch [19–22]. Recent evidences suggest that Hippo pathway coordinates multiple cellular processes involved in immune function [19, 23]. Mst1 and Mst2 regulate immune cell function by promoting cellular adhesion mechanisms, as well as cell-intrinsic regulation of intracellular signaling [20, 21]. In the regulation of immune system homeostasis, Hippo signaling pathway mainly plays a role through non-classical pathways [22]. TLR/MyD88-mediated Mst1/2 activation is necessary for macrophage phagocytic function and effective bacterial clearance [24]. Mst1 acts as a crucial regulator of mitochondrial homeostasis and quiescence in iNKT cell development and effector lineage differentiation [25]. Furthermore, Mst1/2 can stabilize the core transcription factor Foxo1/3 in Treg cells and enhance the activity of STAT5 induced by IL-2, thereby maintaining the survival and proliferation of Treg cells [21]. However, the role of Mst1 and Mst2 in NK cell development, metabolism and effector function is yet to be investigated. Nevertheless, it remains to be explored whether canonical Hippo signaling is required for the prevention of excessive activation of NK cells and maintaining cellular quiescence during their development.

In this study, we show that the development, survival and function of NK cells require the combined action of Mst1 and Mst2, but not other canonical Hippo signaling components. We uncover that Mst1/2 signaling serves as a negative regulator of NK cells in orchestrating their proliferation and mitochondrial homeostasis. Deletion of Mst1/2 at NKp stage significantly impairs NK cell homeostasis in a cell-intrinsic manner, manifested by reduced NK cell pools and weakened functions. Mst1/2-deficient NK cells display heightened mitochondrial respiration, excessive ROS production and disrupted intracellular redox homeostasis, thereby leading to NK cell apoptosis. More persuasively, inhibition of excessive ROS levels significantly enhances the survival of Mst1/2-deficient NK cells. Importantly, we demonstrate that deletion of Mst1/2 impairs the response of NK cells to IL-15-pSTAT3-TCF1 axis. Reconstructing TCF1 expression largely rescues the survival and developmental defect of Mst1/2-deficient NK cells. Collectively, our findings underscore the critical role played by Mst1/2 in metabolic reprogramming and transcriptional regulation for NK cell survival and function, providing insights into immune homeostasis maintenance and immune surveillance.

## Results

### Mst1 and Mst2 deficiency at NKp stage disturbs the homeostasis of NK cells

**Fig. 1:**
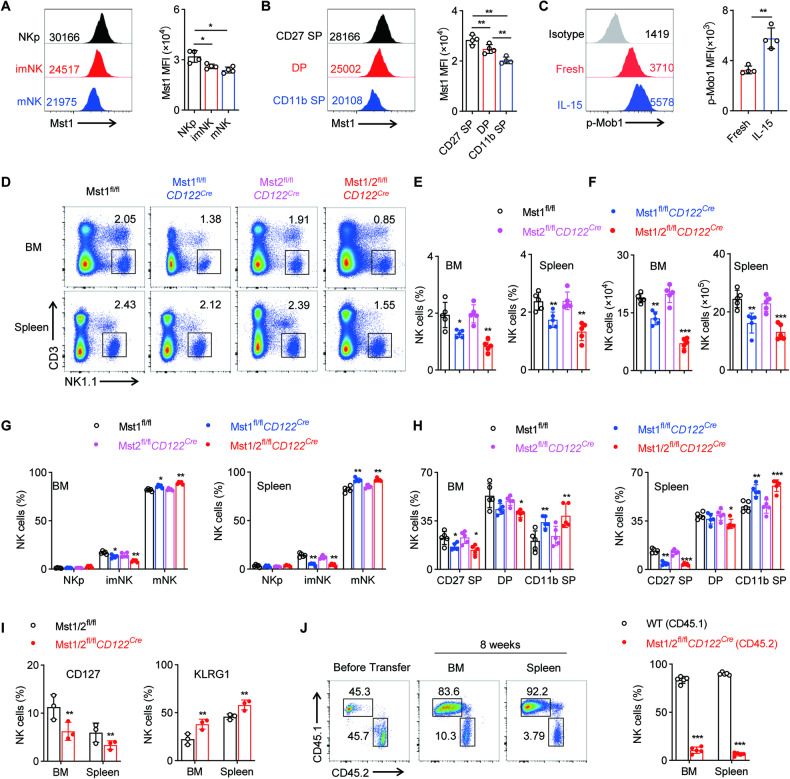
Mst1/2 are indispensable for early NK cell development. **A** Flow cytometry analysis (left) and mean fluorescence intensity (MFI, right) of Mst1 levels in NKp (CD3^−^CD122^+^CD11b^−^NK1.1^−^), immature NK (CD3^−^CD122^+^CD11b^−^NK1.1^+^, imNK) and mature NK (CD3^−^CD122^+^CD11b^+^NK1.1^+^, mNK) cells from the spleen of wild-type mice (WT) (*n* = 4). **B** Flow cytometry analysis (left) and MFI intensity (right) of Mst1 levels in CD27 SP (CD3^−^NKp46^+^CD27^+^CD11b^−^), DP (CD3^−^NKp46^+^CD27^+^CD11b^+^) and CD11b SP (CD3^−^NKp46^+^CD27^−^CD11b^+^) NK cells from the spleen of WT mice (*n* = 4). **C** Flow cytometry analysis (left) and MFI intensity (right) of p-Mob1 expression in splenic NK cells before and after stimulation with 50 ng/mL IL-15 for 30 min (*n* = 4). **D**–**F** Flow cytometry analysis of CD3^−^NK1.1^+^ NK cells in bone marrow (BM) and spleen from WT and the indicated conditional knockout mice (**D**). The percentages (**E**) and the absolute numbers (**F**) of NK cells in spleen and BM from the indicated mice (*n* = 5). The percentages of NKp, imNK, mNK cells on gated CD3^−^CD122^+^ cells (**G**) and CD27 SP, DP, CD11b SP NK cell subsets on gated CD3^−^NKp46^+^ cells (**H**) in BM (left) and spleen (right) from the indicated mice (*n* = 5). **I** Percentages of CD127^+^ (left) and KLRG1^+^ (right) NK cells in the BM and spleen from the Mst1/2^fl/fl^ and Mst1/2^fl/fl^*CD122*^*Cre*^ mice (*n* = 3). **J** Representative plots (left) and summary data (right) of the percentage of NK cells in the BM and spleen from BM chimera mice (*n* = 5). Data of **A**–**I** are representative of three independent experiments with similar results. Data of **J** are representative of two independent experiments with similar results.

To gain a comprehensive understanding of the regulation and requirement of Hippo signaling during NK cell development, we first examined the expression profile of Mst1 in NK cells at different developmental stages. Our analysis revealed a dynamic pattern of Mst1 expression, characterized by particularly high levels at the early stage of NK cell development. Specifically, the expression level of Mst1 was found to be higher in NKp (CD3^–^CD122^+^NK1.1^–^CD11b^–^) cells compared to that in immature (CD3^–^CD122^+^NK1.1^+^CD11b^–^, imNK) and mature (CD3^–^CD122^+^NK1.1^+^CD11b^+^, mNK) NK cells (Fig. 1A). Furthermore, CD27^+^CD11b^–^ (CD27 SP) immature NK cells exhibited higher Mst1 expression than CD27^–^CD11b^+^ (CD11b SP) terminal mature NK cells (Fig. 1B). Upon IL-15 stimulation, the phosphorylation of Mob1, a well-established downstream target of Mst1/2, was observed to increase in NK cells (Fig. 1C). These findings suggest potential involvement of the Hippo signaling pathway in early stage of NK cell development.

To better understand the role of Hippo signaling in early NK cell development, we generated mice with targeted deletion of key molecules in this pathway. Specifically, we crossed mice carrying floxed alleles for Mst1 (encoded by *Stk4*) and Mst2 (encoded by *Stk3*) with *CD122*^*Cre*^ mice to generate mice with specific deletions of Mst1 and/or Mst2 at the NKp stage (denoted Mst1^fl/fl^*CD122*^*Cre*^, Mst2^fl/fl^*CD122*^*Cre*^ or Mst1/2^fl/fl^*CD122*^*Cre*^) (Fig. S1A, B). Although CD122 is expressed on some T cells, our findings showed that the deletion of either Mst1 or Mst2 alone, as well as the combined deficiency of Mst1 and Mst2 in CD122^+^ cells, had no impact on the development of CD4^+^, CD8^+^ T and Treg cells (Fig. S1C–G). However, in the absence of Mst1, there were significant decreases in the frequencies and numbers of NK cells in bone marrow (BM) and spleen compared with wild-type (WT) mice (Fig. 1D–F). In the BM and spleen, Mst1 deficiency altered the developmental stages of NK cells, resulting in a reduced frequency of imNK cells and an increased frequency of mNK cells, while no change was observed in NKp cell population (Fig. 1G, Fig. S2A, B). Furthermore, based on CD27 and CD11b expression levels, we observed notable decreases in the frequencies of CD27^+^CD11b^−^ (CD27 SP) and CD27^+^CD11b^+^ (DP) NK cell subsets but an enrichment of CD27^−^CD11b^+^ (CD11b SP) terminal mNK cells in Mst1-deficient mice (Fig. 1H, Fig. S2C, D). Unlike Mst1, the genetic deletion of Mst2 alone did not significantly diminish the frequency or quantity of NK cells both in the BM and spleen (Fig. 1D–F). Additionally, mice lacking Mst2 alone exhibited normal frequencies and numbers of NK cell developmental stages (Fig. 1G, H, Fig. S2A–D). Previous studies have suggested a genetic redundancy between Mst1 and Mst2 [26]. Consistent with this hypothesis, simultaneous deletion of both Mst1 and Mst2 (Mst1/2) resulted in a trend towards further reduction in the frequency and number of NK cells than deletion of Mst1 alone (Fig. 1D–F). Furthermore, although the numbers of all NK cell subsets were decreased, Mst1/2-deficent mice displayed elevated frequency of mature NK cells but reduced proportion of immature NK cells compared to those lacking Mst1 alone, indicating a more severe impairment in NK cell differentiation (Fig. 1G, H, Fig. S2A–D). Additionally, the expression of immature markers CD127 was reduced, while the terminal maturation receptor KLRG1 [27, 28] was significantly increased in NK cells from BM and spleen of Mst1/2-deficient mice (Fig. 1I). These findings highlight the important role played by Mst1 and Mst2 in maintaining homeostasis and facilitating development of NK cells.

To investigate the intrinsic requirement of Mst1/2 in NK cell development, we generated BM chimeric mice by mixing BM cells from WT control (CD45.1) and Mst1/2^fl/fl^*CD122*^*Cre*^ mice (CD45.2) at a 1:1 ratio, which were then transplanted into sub-lethally irradiated CD45.1^+^CD45.2^+^ hosts for an 8-week period (Fig. S2E). The relative proportion of NK cells derived from Mst1/2^fl/fl^*CD122*^*Cre*^ mice exhibited significant impairment compared to those derived from WT mice (Fig. 1J). These findings strongly suggest that Mst1 and Mst2 play a cell-intrinsic role in regulating NK cell homeostasis.

### Deletion of Mst1/2 severely compromises NK cell function

**Fig. 2:**
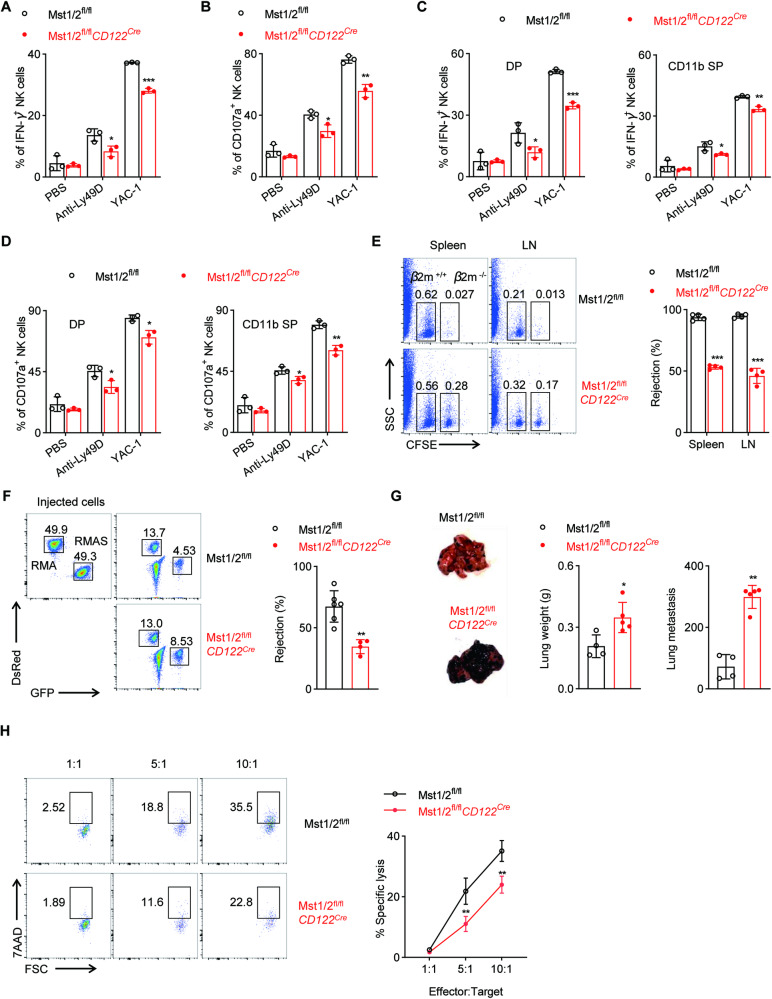
The deficiency of Mst1/2 at NKp stage impairs NK cell function. Expression levels of IFN-γ (**A**) and CD107a (**B**) in total NK cells from spleen of Mst1/2^fl/fl^ and Mst1/2^fl/fl^*CD122*^*Cre*^ mice after stimulation with anti-Ly49D antibody or YAC-1 cells (*n* = 3). Expression levels of IFN-γ (**C**) and CD107a (**D**) in CD27^+^CD11b^+^ DP (left) and CD27^−^CD11b^+^ CD11b SP (right) subsets of NK cell from spleen of Mst1/2^fl/fl^ and Mst1/2^fl/fl^*CD122*^*Cre*^ mice after stimulation with anti-Ly49D antibody or YAC-1 cells (*n* = 3). **E** Representative flow cytometry plots (left) and the percentage (right) of rejected *β2m*^*−/−*^ splenocytes cells in the spleen and lymph nodes (LN) from Mst1/2^fl/fl^ and Mst1/2^fl/fl^*CD122*^*Cre*^ mice (*n* = 4). **F** Representative flow cytometry plots (left) and the percentage (right) of rejected RMA-S cells from Mst1/2^fl/fl^ and Mst1/2^fl/fl^*CD122*^*Cre*^ mice (*n* ≥ 4). **G** Representative images (left), the lung weights (middle) and the number of tumor nodules (right) from Mst1/2^fl/fl^ and Mst1/2^fl/fl^*CD122*^*Cre*^ mice (*n* ≥ 4). **H** FACS analysis of the cytotoxicity of NK cells from Mst1/2^fl/fl^ and Mst1/2^fl/fl^*CD122*^*Cre*^ mice against RMA-S cells was detected by 7AAD staining at different *E*:*T* ratios (*n* = 4). Data are representative of three independent experiments with similar results.

The maturation of NK cells is accompanied by an enhancement of effector functions. Therefore, we proceeded to evaluate the functional effects of Mst1/2-deficient NK cells both in vitro and in vivo. The residual NK cells in Mst1/2^fl/fl^*CD122*^*Cre*^ mice exhibited a severe impairment in IFN-γ production and CD107a expression following stimulation with anti-Ly49D antibody or YAC-1 cells (Fig. 2A, B, Fig. S3A, B). This impairment of NK cell function resulting from Mst1/2 deletion is not attributed to developmental obstruction, as evidenced by the further analysis of functional sub-populations, which also revealed an impediment in IFN-γ production and CD107a expression both in DP and CD11b SP NK cell subsets after Mst1/2 deletion (Fig. 2C, D, Fig. S3C–F). We then evaluated the capacity of NK cells to mediate “missing-self” rejection and found that Mst1/2 deficiency in NK cells compromised clearance of MHC-I deficient splenocytes (Fig. 2E). Similarly, substantial defects were observed in the ability of Mst1/2-deficient NK cells to eliminate target RMA-S tumor cells (Fig. 2F). Furthermore, using a B16F10 lung metastasis tumor model, we discovered that compared to WT control mice, Mst1/2-deficient mice displayed significantly increased lung weight and tumor colony numbers (Fig. 2G). To further validate the compromised functionality of Mst1/2-deficient NK cells, we conducted in vitro co-culture experiments using comparable NK cells isolated from Mst1/2^fl/fl^ or Mst1/2^fl/fl^*CD122*^*Cre*^ mice at varying ratios with RMA-S cells. The results demonstrated that NK cells lacking Mst1/2 exhibited diminished cytotoxic capacity compared to wild-type NK cells (Fig. 2H). Overall, these findings reveal that Mst1/2 play an important role in the function of NK cells.

### Mst1/2 regulate the development and function of NK cells independent of canonical Hippo targets

**Fig. 3:**
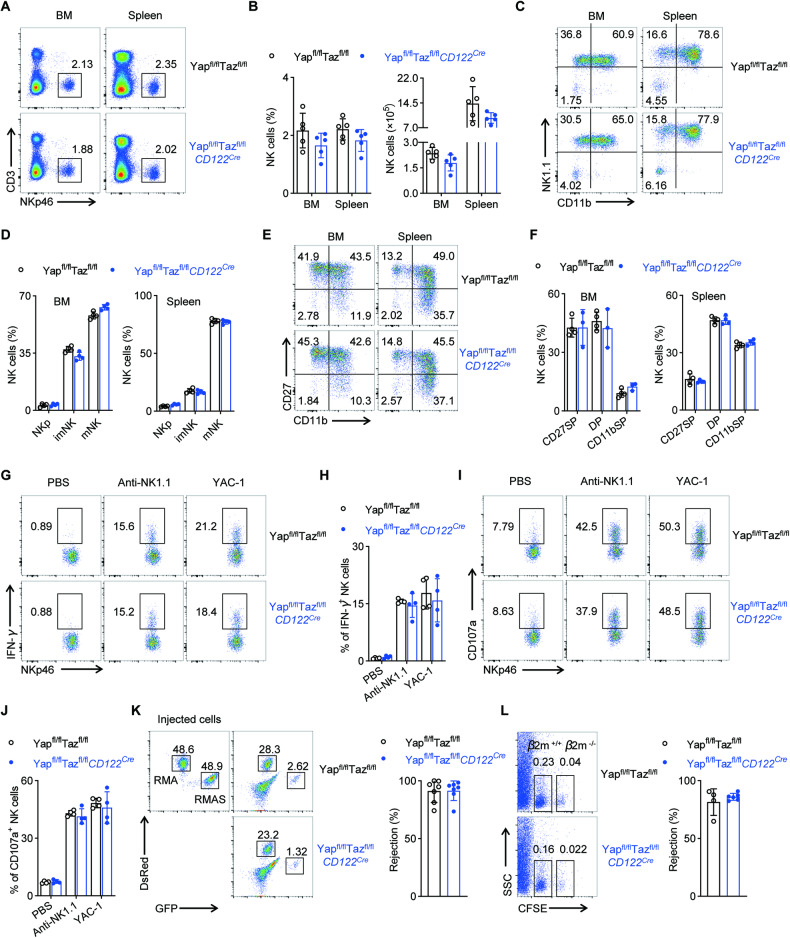
Loss of Yap and Taz has not effect on NK cell development and function. Representative plots (**A**) and quantification of the percentage (**B**, left) and absolute number (B, right) of NK cells in BM and spleen from Yap^fl/fl^Taz^fl/fl^ and Yap^fl/fl^Taz^fl/fl^*CD122*^*Cre*^ mice (*n* = 5). Representative plots (**C**) and quantification of the percentage of NKp, imNK, mNK cells among gated CD3^−^CD122^+^ cells in BM (**D**, left) and spleen (**D**, right) from Yap^fl/fl^Taz^fl/fl^ and Yap^fl/fl^Taz^fl/fl^*CD122*^*Cre*^ mice (*n* = 4). Representative plots (**E**) and quantification of the percentage of CD27 SP, DP, CD11b SP NK cell subsets among gated CD3^−^NKp46^+^ cells in BM (**F**, left) and spleen (F, right) from Yap^fl/fl^Taz^fl/fl^ and Yap^fl/fl^Taz^fl/fl^*CD122*^*Cre*^ mice (*n* = 4). Representative plots (**G**) and the statistical chart (**H**) show the percentages of IFN-γ^+^ NK cells from spleen of Yap^fl/fl^Taz^fl/fl^ and Yap^fl/fl^Taz^fl/fl^*CD122*^*Cre*^ mice (*n* = 4). Representative plots (**I**) and the statistical chart (**J**) show the percentages of CD107a^+^ NK cells from spleen of Yap^fl/fl^Taz^fl/fl^ and Yap^fl/fl^Taz^fl/fl^*CD122*^*Cre*^ mice (*n* = 4). **K** Representative flow cytometry plot (right) and the percentage (left) of rejected RMA-S cells from Yap^fl/fl^Taz^fl/fl^ and Yap^fl/fl^Taz^fl/fl^*CD122*^*Cre*^ mice (*n* = 7). **L** Representative flow cytometry plot (left) and the percentage (right) of rejected *β2m*^*-/-*^ splenocytes cells in the spleen from Yap^fl/fl^Taz^fl/fl^ and Yap^fl/fl^Taz^fl/fl^*CD122*^*Cre*^ mice (*n* ≥ 4). Data of **A**–**J** are representative of three independent experiments with similar results. Data of **K** and **L** are representative of two independent experiments with similar results.

In the canonical Hippo signaling pathway, the nuclear co-factors Yap (encoded by *Yap1*) and Taz (encoded by *Wwtr1*) mediate Mst1/2 signaling [29]. To determine if the canonical Hippo pathway is required for NK cell development and function, we crossed *CD122*^*Cre*^ mice with Yap^fl/fl^ and Taz^fl/fl^ mice (denoted Yap^fl/fl^Taz^fl/fl^*CD122*^*Cre*^). Yap/Taz deficiency did not affect the frequencies and numbers of NK cells and their developmental stages (Fig. 3A–F). Moreover, we found that NK cells from the Yap/Taz-deficient mice held comparable IFN-γ production and CD107a expression when compared to those from WT mice (Fig. 3G–J). At the same time, just like the control mice, the Yap/Taz-deficient mice also successfully removed the RMA-S cells and eliminated the mismatched *β2m*^*-/-*^ splenocytes (Fig. 3K, L). Therefore, it appears that Yap/Taz is not required for the development and function of NK cells, implying that Mst1/2 regulate NK cell development and function through a noncanonical Hippo signaling pathway.

### Mst1/2 enforce NK cell quiescence and viability

**Fig. 4:**
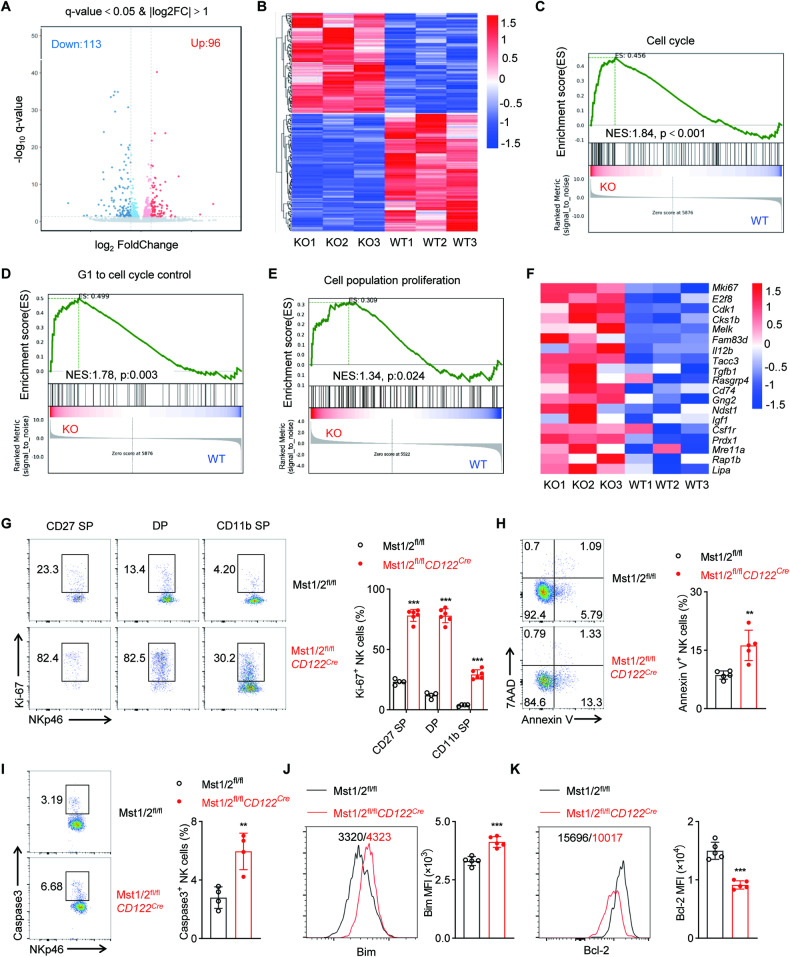
Mst1/2 coordinate the cellular quiescence of NK cells with survival. **A**–**F** RNA sequencing was performed on NK cells sorted from splenocytes in Mst1/2^fl/fl^ and Mst1/2^fl/fl^*CD122*^*Cre*^ mice. **A** The volcano plot illustrates genes that exhibit differential expression between NK cells from Mst1/2^fl/fl^ and Mst1/2^fl/fl^*CD122*^*Cre*^ mice, with lines indicating a twofold difference (edgeR, *p* < 0.05). **B** The heatmap displays the expression levels of up- and downregulated genes in Mst1/2-depleted NK cells compared to control NK cells. Gene-set enrichment analysis of RNA-seq data reveals an enrichment of the related genes for cell cycle (**C**), G1 to cell cycle control (**D**) and cell population proliferation (**E**) in Mst1/2-deficient NK cells compared to WT NK cells. **F** Heatmap analysis of RNA-seq data demonstrates differentially expressed cell proliferation-related genes between NK cells from Mst1/2^fl/fl^ and Mst1/2^fl/fl^*CD122*^*Cre*^ mice. **G** Representative plots (left) and statistic data (right) illustrate Ki-67^+^ NK cell subsets in spleen from Mst1/2^fl/fl^ and Mst1/2^fl/fl^*CD122*^*Cre*^ mice (*n* ≥ 4). Representative plots (left) and statistic data (right) illustrate Annexin V^+^ (**H**) and active caspase3^+^ (**I**) NK cells in spleen from Mst1/2^fl/fl^ and Mst1/2^fl/fl^*CD122*^*Cre*^ mice (*n* ≥ 4). Flow cytometry analysis (left) and MFI intensity (right) show Bim (**J**) and Bcl-2 (**K**) levels in splenic NK cells from Mst1/2^fl/fl^ and Mst1/2^fl/fl^*CD122*^*Cre*^ mice (*n* = 5). Data of **G**–**K** are representative of three independent experiments with similar results.

To comprehensively investigate the effects of Mst1/2 deficiency on early NK cell homeostasis, we conducted global RNA sequencing (RNA-seq) analysis of sorted total NK cells from spleen of Mst1/2^fl/fl^ and Mst1/2^fl/fl^*CD122*^*Cre*^ mice. The result revealed 96 genes up-regulated and 113 genes down-regulated in Mst1/2-deficient NK cells, exhibiting fold changes greater than 2 (Fig. 4A, B). Subsequently, gene-set enrichment analysis (GSEA) was performed on the RNA-seq data to elucidate the underlying biological processes. The results demonstrated a significant enrichment of pathways related to cell cycle regulation and cellular proliferation in NK cells from Mst1/2^fl/fl^*CD122*^*Cre*^ mice (Fig. 4C–F). Consistent with this observation, deletion of Mst1 and Mst2 together resulted in a significant increase in Ki-67 expression across all subsets of NK cells compared to WT NK cells (Fig. 4G). These findings suggest that the decrease in NK cell numbers observed upon loss of Mst1/2 is not due to impaired proliferation. Subsequently, we assessed the level of apoptosis in WT and Mst1/2^fl/fl^*CD122*^*Cre*^ mice. Notably, we observed an elevated proportion of apoptotic cells characterized by higher Annexin V abundance in Mst1/2-deficient NK cells (Fig. 4H). In addition, pro-apoptotic molecules, including active caspase3 and Bim, were significantly elevated in Mst1/2-deficient NK cells (Fig. 4I, J). Moreover, compared with WT counterparts, there was a considerable decrease in the expression of anti-apoptotic protein Bcl-2 in Mst1/2-deficient NK cells (Fig. 4K). Collectively, these findings demonstrate that Mst1/2 enforce the establishment of cellular quiescence and cell viability for NK cells.

### Mst1/2 control metabolic homeostasis to maintain NK cell survival

**Fig. 5:**
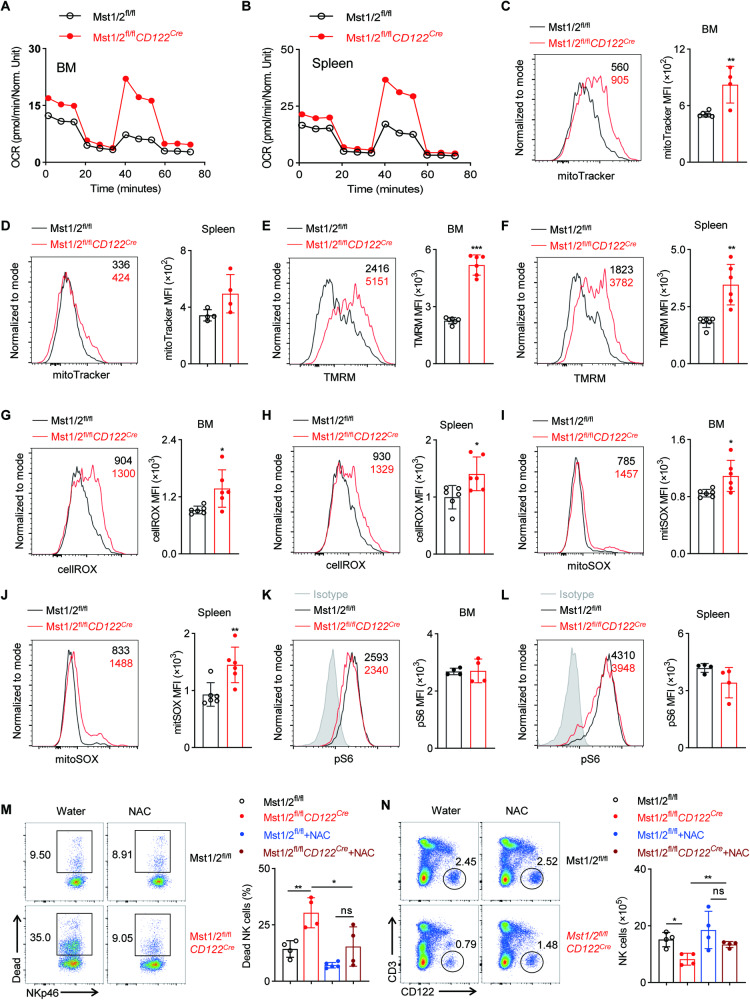
Mst1/2 control mitochondrial metabolism and redox homeostasis in NK cells. Seahorse extracellular flux analysis was performed to measure the oxygen consumption rate (OCR) in NK cells from the BM (**A**) and spleen (**B**) of Mst1/2^fl/fl^ and Mst1/2^fl/fl^*CD122*^*Cre*^ mice. Flow cytometry analysis (left) and MFI intensity (right) illustrate the expression levels of mitoTracker (**C**, **D**), TMRM (**E**, **F**), total cellular ROS (**G**, **H**), mitochondrial ROS (**I,** **J**) and p-S6 (**K**, **L**) in NK cells from BM (**C**, **E**, **G**, **I**, **K**) and spleen (**D**, **F**, **H**, **J**, **L**) of Mst1/2^fl/fl^ and Mst1/2^fl/fl^*CD122*^*Cre*^ mice (*n* ≥ 4). **M** Representative flow cytometry plot (left) and summary data (right) display the percentage of dead cells in spleen from Mst1/2^fl/fl^ and Mst1/2^fl/fl^*CD122*^*Cre*^ mice fed water with or without NAC (1.5 g/L) for 30 days starting at 21-day-old (*n* ≥ 4). **N** Representative flow cytometry plot (left) and summary data (right) show the number of CD3^−^CD122^+^ cells in spleen from Mst1/2^fl/fl^ and Mst1/2^fl/fl^*CD122*^*Cre*^ mice fed water with or without NAC (1.5 g/L) for 30 days starting at 21-day-old (*n* ≥ 4). Data of **C**–**L** are representative of three independent experiments with similar results. Data of M and N are representative of two independent experiments with similar results.

Mst1 plays a crucial role in regulating metabolic and mitochondrial homeostasis during the development of iNKT cells and macrophages [25, 30]. As Mst1/2-deficient NK cells exhibited reduced quiescence features, we next investigated whether these defects were associated with alterations in cellular metabolism. Therefore, we conducted measurement of the oxygen consumption rate (OCR) and observed an elevation of OCR in Mst1/2-deficient NK cells compared to WT NK cells (Fig. 5A, B), indicating aberrant increases in mitochondrial metabolism. Subsequently, we characterized the mitochondrial features and noted elevated MitoTracker and tetramethylrhodamine, methyl ester (TMRM) staining in Mst1/2-deficient NK cells when compared to WT NK cells (Fig. 5C–F), which aligns with the elevated OCR. Furthermore, intracellular reactive oxygen species (ROS) and mitochondrial ROS levels were found to be up-regulated in Mst1/2-deficient NK cells compared to the control group (Fig. 5G–J). It is worth mentioning that while mTORC1 signaling plays a role in promoting mitochondrial metabolism and NK cell proliferation [31, 32], our findings demonstrated slightly reduced mTORC1 signaling, as determined by the phosphorylation of S6, in Mst1/2-deficient NK cells (Fig. 5K, L). This suggests that the observed enhancements in mitochondrial parameters and cell proliferation in Mst1/2-deficient NK cells are unlikely to be attributed to heightened mTORC1 activity. It is widely recognized that maintaining an appropriate level of ROS is crucial for cellular function [33–35]. However, excessive accumulation of ROS can have detrimental effects on cell survival. In order to investigate the potential contribution of elevated ROS levels in Mst1/2-deficient NK cells to their decreased viability, we administered the ROS scavenger N-acetyl-L-cysteine (NAC) into Mst1/2^fl/fl^*CD122*^*Cre*^ and Mst1/2^fl/fl^ mice via drinking water. Remarkably, the administration of NAC significantly mitigated NK cell death and increased the number of NK cells in Mst1/2^fl/fl^*CD122*^*Cre*^ mice (Fig. 5M, N). These findings suggests that the excessive accumulation of ROS is closely associated with the impaired survival of Mst1/2-deficient NK cells. Taken together, these results indicate that Mst1/2 primarily regulates mitochondrial homeostasis, which in turn affects the establishment of quiescence program that is necessary for the maintenance of NK cell survival.

### Mst1/2 regulate NK cell development and homeostasis dependent on TCF1

**Fig. 6:**
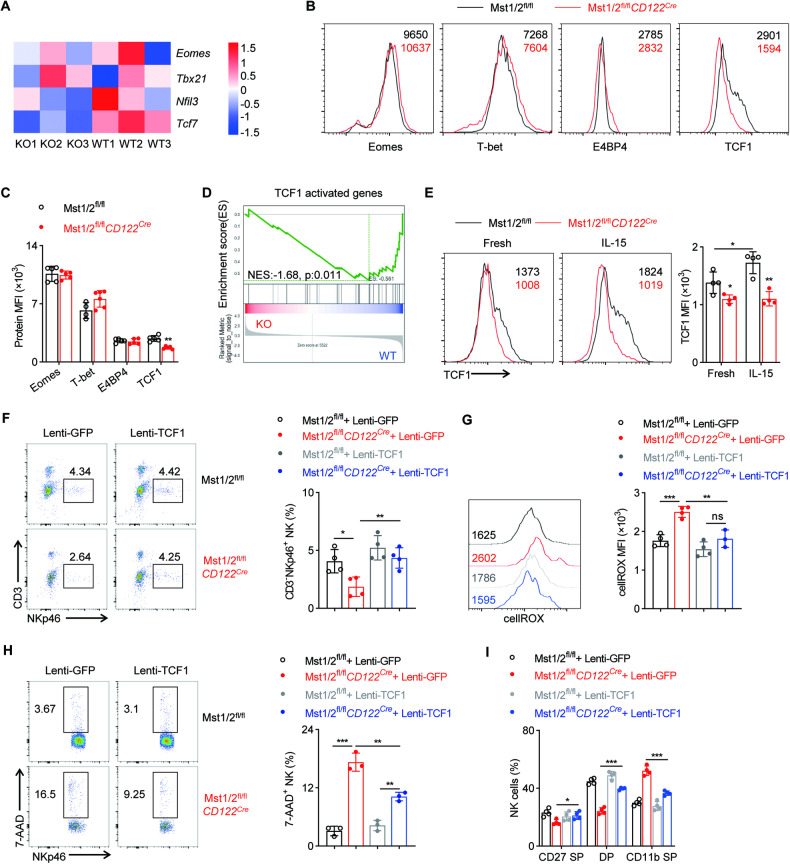
Mst1/2 program TCF1 expression to maintain NK cell homeostasis. **A** Heatmap analysis of RNA-seq data reveals differential expression of certain transcription factors between NK cells from Mst1/2^fl/fl^ and Mst1/2^fl/fl^*CD122*^*Cre*^ mice. Flow cytometry analysis (**B**) and MFI intensity (**C**) demonstrate the levels of Eomes, T-bet, E4BP4 and TCF1 in splenic NK cells from Mst1/2^fl/fl^ and Mst1/2^fl/fl^*CD122*^*Cre*^ mice (*n* ≥ 4). **D** Gene-set enrichment analysis of RNA-seq data indicates a negative enrichment of TCF1-activated genes in Mst1/2-deficient NK cells compared to WT NK cells. **E** Flow cytometry analysis (left) and MFI intensity (right) reveal the levels of TCF1 in NK cells from the spleen of Mst1/2^fl/fl^ and Mst1/2^fl/fl^*CD122*^*Cre*^ mice upon stimulation with or without IL-15 (10 ng/mL) for 16 h (*n* = 4). **F**–**I** Representative plots (left panel) and summary data (right panel) illustrate the percentage of NK cells (**F**), the MFI of total cellular ROS levels (**G**), the percentage of 7AAD^+^ NK cells (H) and distribution pattern of different NK cell subsets (I) in the spleen of BM chimeric mice (*n* ≥ 3). Data **B**, **C** and **E** are representative of three independent experiments with similar results. Data **F**–**I** are representative of two independent experiments with similar results.

We further investigated the specific Hippo targets within NK cells. The development and survival of NK cells are tightly regulated by various transcription factors, including Eomes, T-bet, E4BP4 and TCF1. The deletion of Mst1/2 did not affect the expression of Eomes, T-bet and E4BP4 in NK cells (Fig. 6A–C). However, we identified a significant down-regulation of the transcriptional level of *Tcf7* in Mst1/2-deficient NK cells (Fig. 6A). Flow cytometric analyses confirmed the decreased expression of TCF1 (encoded by *Tcf7*) in Mst1/2-deficieint NK cells (Fig. 6B, C). TCF1 has been reported to play a crucial role in promoting normal NK cell development and function by sustaining the expression of multiple regulators including Gzmb, Ets1, etc [36, 37]. To assess whether TCF1 function is defective in Mst1/2-deficient NK cells, we performed Gene-Set Enrichment Analysis (GSEA) using a set of genes well-known to be activated by TCF1 in NK cells [36]. Interestingly, the enrichment analysis revealed a significant enrichment of TCF1-activating genes in WT NK cells compared to Mst1/2-deficient NK cells (Fig. 6D), suggesting the impaired activation of TCF1 in Mst1/2-deficient NK cells. Moreover, IL-15 was found to activate TCF1 expression specifically in WT but not in Mst1/2-deficient NK cells (Fig. 6E), suggesting Mst1/2 mediate the regulation of IL-15 on TCF1 activation. Subsequently, we aimed to investigate whether the developmental deficiency caused by Mst1/2 deletion could be rescued by introducing TCF1. To address this question, we employed a retroviral system for ectopically express TCF1 in hematopoietic stem cells (HSCs) derived from Mst1/2^fl/fl^*CD122*^*Cre*^ and control mice, and subsequently transplanted these modified HSCs into irradiated host CD45.1^+^CD45.2^+^ mice. After an eight-week period post-transplantation, we observed a substantial increase in the proportion of TCF1 over-expressing NK cells in Mst1/2^fl/fl^*CD122*^*Cre*^ mice (Fig. 6F). The subsequent analysis revealed that TCF1 improved the viability of Mst1/2-deficient NK cells by attenuating the intracellular ROS concentration, thereby augmenting the population of NK cells (Fig. 6G, H). The defects in NK cell development caused by Mst1/2 deletion were also improved after TCF1 over-expression (Fig. 6I). Collectively, aforementioned results confirm that TCF1 is indispensable for Mst1/2 in maintaining NK cell homeostasis.

### Mst1/2 regulate *Tcf7* gene transcription by promoting STAT3 phosphorylation

**Fig. 7:**
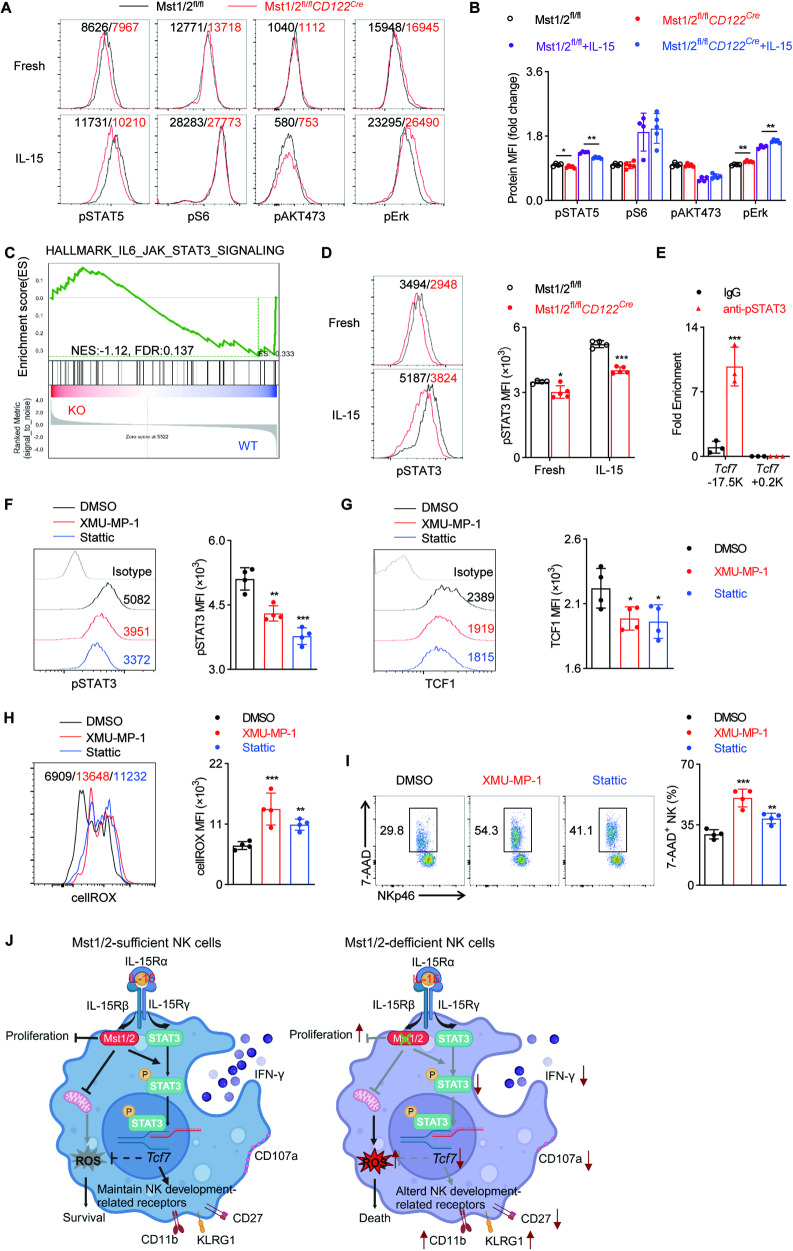
Mst1/2 regulate TCF1 expression by promoting the phosphorylation of STAT3. **A**, **B** Flow cytometry analysis (left) and MFI intensity (right) demonstrate the expression levels of p-STAT5, p-S6, p-AKT473 and p-Erk in NK cells from Mst1/2^fl/fl^ and Mst1/2^fl/fl^*CD122*^*Cre*^ mice upon stimulation with or without IL-15 (50 ng/mL) for 30 min (*n* ≥ 4). **C** Gene-set enrichment analysis of RNA-seq data shows the enrichment of hallmark-IL6-JAK-STAT3-signaling related genes in Mst1/2-deficient NK cells compared to WT NK cells. **D** Flow cytometry analysis (left) and MFI intensity (right) reveals the expression level of p-STAT3 (Ser727) in NK cells from Mst1/2^fl/fl^ and Mst1/2^fl/fl^*CD122*^*Cre*^ mice upon stimulation with or without IL-15 (50 ng/mL) for 30 min (*n* = 4). **E** ChIP-qPCR analysis demonstrated binding of p-STAT3 conserved motifs in the *Tcf7* 5′ regulatory region (−17.5 kb), while no binding was observed in a region without p-STAT3-binding motifs (+0.2 kb), serving as a negative control. The experiments were using sorted NK cells from WT mice with anti-p-STAT3 antibody or isotype-matched IgG. **F**–**I** WT splenocytes were treated with DMSO, Mst1 inhibitor XMU-MP-1 (1 mM, MedChemExpress) or p-STAT3 inhibitor Stattic (1 mM, MedChemExpress) for 24 h, followed by flow cytometry analysis. The representative plots (left) and summary data (right) show the expression level of p-STAT3 (F), TCF1 (**G**), total cellular ROS (**H**) and the percentage of 7AAD^+^ NK cells (**I**) (*n* = 4). **J** Schematic illustration of Mst1/2 regulate NK cell survival and function via controlling metabolic state and transcriptional activity. Data **A**, **B** and **D**–**I** are representative of three independent experiments with similar results.

In order to gain insight into the molecular mechanisms underlying the altered IL-15 response of Mst1/2-deficient NK cells, we investigated several well-known IL-15-dependent signaling pathways, including the JAK-STAT5 pathway, mTOR signaling pathways and extracellular signal-regulated kinases (ERKs) signaling pathways [38–41]. We observed only a slight reduction in STAT5 phosphorylation in Mst1/2-deficient NK cells compared to WT NK cells with or without IL-15 stimulation. Additionally, the phosphorylation levels of S6 (a target for mTORC1) and AKT473 (a target for mTORC2) remained unchanged while ERK phosphorylation was even increased (Fig. 7A, B). These findings suggest that Mst1/2 in NK cells may respond to the IL-15 signaling pathway through distinct sets of downstream signaling cascades. From the GSEA of the RNA-seq data, we observed a significantly negative enrichment of the “IL6_JAK_STAT3_SIGNALING” pathway in NK cells of Mst1/2^fl/fl^*CD122*^*Cre*^ mice (Fig. 7C). Consistently, Mst1/2-deficient NK cells exhibited markedly reduced levels of STAT3 phosphorylation, particularly upon IL-15 stimulation (Fig. 7D). STAT3 has been reported to be downstream of IL-15 receptor signaling [42]. These results imply that STAT3 likely functions downstream of endogenous IL-15 to coordinate Mst1/2-mediated regulation of NK cell development.

The previous studies have demonstrated the direct binding of pSTAT3 to *Tcf7* promoter, regulating its expression in follicular regulatory T (Tfr) cells [43]. Our findings indicate that Mst1/2 modulates NK cell development by influencing the expression of TCF1. We subsequently investigated whether the phosphorylation of STAT3, mediated by Mst1/2, governed the regulation of TCF1 during NK cell differentiation. Through the implementation of chromatin immunoprecipitation (ChIP)-qPCR experiments utilizing sorted NK cells, we heightened affinity between p-STAT3 and the sequences (TTCnnnGAA) located in the 5′-regulatory region of *Tcf7* (−17.5 kb), as compared to the binding exhibited by isotype-control IgG or p-STAT3’s interaction with the first exon (*Tcf7* + 0.2 kb), which lacked a p-STAT3-binding motif (Fig. 7E). It is suggested that pSTAT3 can also bind to *Tcf7* locus in NK cells, thereby regulating the expression of TCF1. To further elucidate the regulation of IL-15-Mst1-pSTAT3-TCF1, we next stimulated wild-type NK cells with IL-15 in the presence or absence of the Mst1 inhibitor XMU-UP-1 or the pSTAT3 inhibitor Stattic. In line with the genetic approach, we observed a severe impairment in STAT3 phosphorylation and TCF1 expression upon inhibition of Mst1 (Fig. 7F, G). Additionally, inhibition of pSTAT3 also significantly impaired TCF1 expression (Fig. 7F, G). Furthermore, both Mst1 and pSTAT3 inhibition led to increased ROS production and cell death in NK cells (Fig. 7H, I). Collectively, these findings highlight the primary role of the IL-15-Mst1/2-pSTAT3-TCF1 axis in regulating NK cell homeostasis.

## Discussion

Metabolic pathways play crucial roles in NK cell development and function. Our group previously reported that optimal mTOR signaling pathway is critical for NK cell differentiation and activation as a central regulator of cell metabolism [14, 16, 32, 40, 44]. However, the exploration of whether additional signaling pathways are required to prevent excessive activation of NK cells and maintain their cellular quiescence during development remains limited. In this study, we revealed the role of Hippo kinases Mst1 and Mst2 in maintaining NK cell homeostasis (Fig. 7J). Loss of Mst1/2 impairs NK cell development, survival and function in a canonical Hippo signaling independent mode. The proper function of Mst1/2 is essential for inducing cell quiescence in NK cells by inhibiting proliferation and mitochondrial metabolism to ensure their development and survival. Furthermore, Mst1/2 can regulate the transcriptional activity by facilitating the activation of pSTAT3-TCF1, which contributes to NK cell homeostasis.

More and more evidence demonstrates the indispensable role of the Hippo signaling pathway in regulating immune function and maintaining immune homeostasis. While the canonical Mst1/2-Lats1/2-Yap/Taz Hippo signaling cassette primarily controls tissue growth during development and regeneration, studies on the Hippo signaling pathway in immune regulation have predominantly focused on the core Mst1/2 kinases. These kinases directly phosphorylate and activate critical signaling pathways in specific immune cell types, thereby interacting with and regulating other key pathways. The contribution of the canonical or non-canonical Hippo signaling pathway in regulating NK cell development and function remains unclear. In this study, we identified that deficiency of Mst1 alone or combined with Mst2 significantly impairs NK cell survival and development. However, deletion of the transcriptional co-activator Yap/Taz, which is downstream of the canonical Hippo pathway, has no effect on NK cell development and tumor-killing function. This suggests that Mst1/2 are important for the maintenance of NK cell homeostasis, and they act in a non-canonical Hippo signaling dependent manner.

As the primary defenders of host immunity, NK cells efficiently exert cytotoxic effects on virus-infected cells, aging damaged cells and malignant transformed cells. The functionality of NK cells is closely associated with numerous intracellular metabolic signaling pathways, such as mTOR signaling, AMPK signaling and ERK signaling [10, 41, 45–47]. The Hippo signaling pathway has been identified as a critical regulator for immune cell function. For instance, Mst1/2 play a crucial role in orchestrating Treg cell function and survival through an IL-2-pSTAT5 axis dependent manner [21]. Additionally, Mst1/2 coordinate the immune priming function of CD8α^+^ dendritic cells with metabolic fitness [19]. Our findings further confirm that Mst1/2-deficient NK cells exhibit impaired capacity against sensitive tumor cells and MHC-I-deficient target cells in vivo. Furthermore, Mst1/2-deficient NK cells exhibit lower production of IFN-γ and reduced expression of CD107a when stimulated with Ly-49D and YAC-1 cells compared to WT NK cells. Interestingly, the double knockout of Yap/Taz at the NKp stage does not affect NK cell function at all. Consequently, we conclude that Mst1/2 participate in the regulation of NK cell function through a canonical Hippo signaling independent mechanism.

The dynamic regulation of entry and exit from cellular quiescence is essential for the survival, activation and function of immune cells [48]. The establishment of cellular quiescence or activation involves changes in nutrient uptake, anabolic metabolism, catabolism and metabolic reprogramming. Hippo signaling pathway plays a crucial role in maintaining the steady state of immune cells, which has been reported to be indispensable for maintaining cellular quiescence in invariant natural killer T (iNKT) cells. In this study, we discover that the deletion of Mst1/2 at the NK progenitor stage disrupts the quiescence of NK cells. This disruption is characterized by enhanced proliferation capacity and increased intracellular reactive oxygen species (ROS) levels. Furthermore, the ablation of Mst1/2-mediated exit from cellular quiescence results in elevated levels of apoptosis in NK cells. Importantly, the removal of excessive ROS through NAC treatment in vivo remarkably restores NK cell survival and homeostasis. These findings demonstrate that Mst1/2 play a significant role in maintaining cellular quiescence in NK cells by inhibiting proliferation and mitochondrial metabolism to ensure their development and survival.

NK cell homeostasis and function largely rely on the coordination of intracellular regulators, particularly transcription factors. Several studies have investigated the roles of various transcription factors in NK cell development and function [16, 37, 49–52]. In this regard, it remains unclear whether Mst1/2 participate in the regulation of NK cell survival and function via interactions with unknown transcription factors. To address this question, we conducted an analysis of our RNA-seq data and observed a significant down-regulation of *Tcf7* (encoding TCF1) in Mst1/2-deficient NK cells compared to WT NK cells. Intriguingly, *Tcf7*^*fl/fl*^*CD122*^*Cre*^ mice exhibit similar phenotypes to our Mst1/2^fl/fl^*CD122*^*Cre*^ mice [36]. Furthermore, the expression of TCF1 in NK cells appears to be triggered by IL-15 signaling. Encouragingly, the reintroduction of TCF1 into Mst1/2-deficient NK cells using retrovirus significantly restores NK cell survival and rectifies the aberrant exit from quiescence. Ye’s group has demonstrated that pSTAT3 directly regulates TCF1 expression in Treg cells [43]. Interestingly, we observed a similar phenomenon in NK cells using Chip-qPCR analysis. Additionally, pSTAT3 is a downstream mediator of IL-15 signaling in NK cells [53], and Mst1/2-deficient NK cells exhibited lower levels of pSTAT3 compared to WT NK cells. Importantly, both genetic ablation and pharmaceutical inhibition of Mst1/2 or pSTAT3 in vitro lead to reduced TCF1 expression in NK cells, accompanied by increased ROS levels and elevated cell death in NK cells. Consequently, our findings confirm that Mst1/2 maintain NK cell survival through the IL-15-pSTAT3-TCF1 axis.

In summary, our study demonstrates that Mst1/2 signaling functions as a molecular rheostat in NK cells via regulating the metabolic state and transcriptional activity, which collectively govern the survival, development and function of NK cells in a canonical Hippo signaling independent manner.

## Materials and methods

### Mice

Mst1^flox/flox^ (*Stk4*^*flox/flox*^), Mst2^flox/flox^ (*Stk3*^*flox/flox*^), Yap^flox/flox^, Taz^flox/flox^, CD45.1 and *β2m*-deficient mice were purchased from Jackson Laboratory. *CD122*^*Cre/+*^ mice were generated in our laboratory [14, 32, 52]. All conditional floxed mice were crossed with *CD122*^*Cre/+*^ mice to obtain mice with specific gene deletion at NKp stage. Gender- and age-matched wild-type controls and conditional knockout mice aged 6 to 10 weeks were used in our experiments. All the mice were C57BL/6 background and maintained in specific pathogen-free animal facilities at Jinan University under controlled temperature (22 ± 1°C) and exposed to a constant 12-hour light/dark cycle. All animal procedures were approved by the Animal Ethics Committee of Jinan University.

### Flow cytometry

Flow cytometry was conducted using the BD FACS Verse™ (a three-laser flow cytometry analyzer, BD Biosciences) or CYTEK NORTHERN LIGHTS (16V-14B-8R, CYTEK). Monoclonal antibodies against mouse CD4 (GK1.5), CD8 (53-6.7), Foxp3 (FJK-16s), CD3 (145-2C11), CD122 (TM-b1), CD127 (A7R34), Bcl-2 (10C4), KLRG1 (2F1), NK1.1 (PK136), NKp46 (29A1.4), CD107a (1D4B), Eomes (Dan11mag), T-bet (4B10), E4BP4 (MABA223), phosphor-AKT1 (Ser473) (SDRNR), phosphor-S6 (cupk43k) and isotype controls were purchased from eBioscience (San Diego, CA). Monoclonal antibodies against mouse CD11b (M1/70), CD45.2 (Ly-5.2), CD45.1(A20), Ki-67 (SolA15), CD27 (LG.3A10), Annexin V (Cat#640941) and IFN-γ (XMG1.2) were purchased from BioLegend (San Diego, CA). Monoclonal antibodies against mouse pSTAT3 S727 (49/p-Stat3), Caspase-3 (Cat#C92-605) and 7AAD (Cat#559925) were purchased from BD Biosciences (Mississauga, Ontario, Canada). Monoclonal antibodies against mouse TCF1 (Cat#9066S), phospho-Mob1 (Thr35) (Cat#8699), Bim (C34C5) Rabbit mAb (Cat#12186), Phospho-Stat5 (Tyr694) (Cat#9359), phospho-p44/42 MAPK (Erk1/2) (Thr202/Tyr204) (Cat#4370S) and polyclonal antibodies against mouse Mst1 (Cat#3682) were obtained from Cell Signaling Technology (Beverly, MA). Dead cells were excluded by using LIVE/DEAD fixable Aqua dead cell stain kit (Cat#L34965, Invitrogen). For the detection of mitochondrial and intracellular total ROS in NK cells, MitoSOX Red (Cat #M36008, Invitrogen) and CellROX Deep Red (Cat #C10422, Invitrogen) were used according to the manufacturer’s instructions. For the measurement of mitochondrial potential and to label mitochondria mass in NK cells, Tetramethylrhodamine (TMRM) (Cat#T668, Invitrogen) and MitoTracker® Mitochondrion-Selective Probes (Cat#M22426, Invitrogen) were used according to the manufacturer’s instructions. For analysis of surface markers, cells were stained in PBS containing 2% (w/v) BSA at room temperature. Intracellular staining was performed using Foxp3/transcription factor staining buffer kit (Cat #00-5523-00, eBioscience) according to the manufacturer’s instructions. Flow cytometry data were analyzed using Flowjo software (BD Biosciences).

### Cell Lines

B16F10 cells were cultured in DMEM (Hyclone) supplemented with 10% FBS (Gibco). YAC-1, RMA-S cells and RMA cells were cultured in RPMI-1640 (Hyclone) supplemented with 10% FBS. All cells were incubated in 37 °C incubator supplied with 5% CO_2_.

### Bone marrow reconstitution

Wild-type mice (CD45.1^+^) and Mst1/2 conditional knockout mice (CD45.2^+^) were injected intravenously with 300 μl 10 mg/mL 5-Fluorouracil (for a 20 g adult mice) to mobilize hematopoietic stem cells [54]. Five days later, the mice were sacrificed. Bone marrow cells were collected and mixed at a 1:1 ratio. The mixture was intravenously administrated into the CD45.1^+^CD45.2^+^ mice, which had been sub-lethally irradiated with 8 Gy. After eight weeks, the development of NK cells derived from Mst1/2 conditional knockout mice (CD45.2^+^) and from WT mice (CD45.1^+^) were detected by flow cytometry. During the process of bone marrow cell reconstruction, the recipient mice were fed antibiotic water (Neomycin,1 mg/ml) twice a week for two weeks.

### Detection of CD107a expression and intracellular staining for IFN-γ

Mice were pre-treated with intraperitoneal injection of poly (I:C) (10 μg/mg) and were sacrificed after 18 h. Poly (I:C)-activated splenocytes (2 × 10^6^) were co-cultured with equal number of YAC-1 tumor cells in a total volume of 500 μL in 24-well plates. For antibody stimulation, 24-well plates were pre-coated with anti-Ly49D antibody at a concentration of 10 μg/mL or with anti-NK1.1 antibody at a concentration of 1 μg/mL overnight. Splenocytes were stimulated with tumor cells or antibodies for 4 h. BD GolgiStop™ reagent (BD Biosciences) was used to inhibit intracellular protein transport, and meanwhile, APC-conjugated anti-CD107a antibody or respective control isotypes were added at the beginning of incubation to detect lysosome synthesis. Four hours after stimulation, cell mixtures were harvested for detection of intracellular IFN-γ and CD107a expression [55].

### In vivo *β2m*^*−/−*^ splenocytes rejection assay

Splenocytes derived from WT mice or *β2m*^*−/−*^ mice were subjected to red blood cell depletion using Ficoll-Hypaque density gradient centrifugation. Subsequently, the splenocytes obtained from wild-type mice and *β2m*^*−/−*^ mice were labeled with 1 μM CFSE and 10 μM CFSE, respectively. These two types of CFSE-labeled splenocytes were mixed at a 1:1 ratio and then intravenously injected into the indicated mice that had been pre-treated with 10 μg/mg poly (I:C) 18 h before. After eight hours, flow cytometry was used to determine the presence of CFSE-high cells in the spleens and lymph nodes. The rejection ratio was calculated based on the following formula: Rejection (%) =100*(*β2m*^*+/+*^-*β2m*^*−/−*^)/(*β2m*^*+/+*^) [55].

### In vivo RMA-S clearance assay

Mice pre-treated with poly (I:C) (10 ug/mg) 18 h before were intraperitoneally injected with a mixture of target cells consisting of NK-sensitive RMA-S cells expressing GFP (10^6^ cells) and NK-non-sensitive RMA cells expressing Ds-Red (10^6^ cells). After an additional 15-hour period post-injection, the mice were euthanized, and cells in the peritoneal cavity were collected and washed with PBS. Flow cytometry was employed to determine the relative percentages of RMA-S and RMA cells. The rejection ratio was calculated based on the following formula: Rejection (%) = 100*(RMA-RMA-S)/(RMA) [56].

### B16F10 lung metastasis tumor model

B16F10 melanoma cells in the logarithmic growth phase were re-suspended in 1× Hank’s Balanced Salt Solution (HBSS) and were intravenously administered into the mice at a dose of 2 × 10^5^ cells per 100 μL. After 14 days, the mice were euthanized, and their lungs were excised and weighed. Subsequently, the numbers of tumor nodules on the lung tissue were meticulously counted using a dissecting microscope [57].

### RNA isolation and RT-qPCR

Total RNA was extracted from splenic NK cells of Mst1/2^fl/fl^ (WT) and Mst1/2^fl/fl^*CD122*^*Cre*^ (cKO) mice and reverse-transcribed with a Takara reverse transcription kit (Cat#RR037Q). The primers for the mouse genes were: *Stk4-F*: ATATCATTCGGCTACGGAAC, *Stk4-R*: GCCTTGATATCTCGGTGTAT. *Stk3-F*: GGGTCCGTTTCAGACATAAT, *Stk3-R*: GGAGAATATTCCCGGCTTTT. The primers for internal control gene *Gapdh* were: *Gapdh-F*: CCAGCTTAGGTTCATCAGGT, *Gapdh-R*: TTGATGGCAACAATCTCCAC. RT-qPCR was done using TB Green Premix Ex Taq (TaKaRa Biotechnology, Shiga, Japan) with BioRad CFX Connect cycler according to manufacturer instructions. The procedure for RT-qPCR as follow: two-step amplification (35 cycles): pre-denaturation (95 °C, 10 min), denaturation (95 °C, 15 s) and annealing/extension (60 °C, 1 min).

### Retroviral transduction and BM chimeras

The Mouse *Tcf7* cDNA was cloned into the pSLenti-EF1-EGFP-P2A-Puro-CMV-MCS-3xFLAG-WPRE plasmid (OBiO). Empty or *Tcf7*-expressing retroviruses were generated in 293T cells using PEI MAX (Polysciences) according to the manufacturer’s instructions. 8-week-old Mst1/2^fl/fl^ (CD45.2) and Mst1/2^fl/fl^*CD122*^*Cre*^ (CD45.2) mice were intravenously injected with 300 μL 10 mg/mL 5-Fluorouracil (5-FU) (Sigma-Aldrich). After 5 days, BM cells were harvested and suspended in 1 mL RPMI 1640 medium containing 20% FBS, 20 ng/mL SCF (Cat#250-03, PeproTech), 20 ng/mL Flt-3 (Cat#250-31, PeproTech), 10 ng/mL IL-3 (Cat#AF-213-13, PeproTech) and 10 ng/mL IL-6 (Cat#216-16, PeproTech). Subsequently, cells were seeded in a 6-well plate and incubated in 37 °C incubator supplied with 5% CO2 for 24 h. After 24 h, 120 μL of viral suspension and polybrene at a final concentration of 8 μg/mL were added to one well of the plate containing 2 mL cell suspension. After a 30-min incubation, cells were spin-infected at 500 × *g* for 90 min at 32 °C and subsequently incubated at 37 °C for a duration of 2–4 h. The process was repeated once by adding an additional 120 μL concentrated retrovirus into the same well as described above. The plate was then returned to the incubator. 2 h later, the cells were harvested and cell numbers were counted. Finally, they were transferred into sub-lethally irradiated CD45.1.2 mice. After eight weeks, the spleen cells were isolated for flow cytometry analysis [16].

### Metabolic studies

The oxygen consumption rate (OCR) was assessed by mitochondrial stress test kit (Cat#103015-100, Agilent Technology) with a XF96 extracellular flux analyzer (Agilent Technology). NK cells were sorted from the bone marrow and spleen of Mst1/2^fl/fl^ and Mst1/2^fl/fl^*CD122*^*Cre*^ mice. Then the cells were seeded onto a 96-well XF plate coated with Cell-Tak (Cat# 354240; Corning) with 2×10^5^/well at 37 °C incubator for 30 min and analyzed by XF96 extracellular flux analyzer later. For the measurement of mitochondrial stress, NK cells were followed by the sequential addition of oligomycin (1 µM), FCCP (1.5 µM), Rotenone/antimycin A (0.5 µM). The data was analyzed by Seahorse Wave Desktop v2.4 software [58].

### ChIP-qPCR

NK cells were sorted from spleen of WT mice using BD FACS Aria II (BD Bioscience). Chromatin immunoprecipitation (Chip) assays were carried out using a NovoNGS® CUT&Tag® 3.0 High-Sensitivity Kit (Cat#N259-YH01, novoprotein) according to the manufacturer’s instructions [59]. Chromatin fragments were immunoprecipitated with anti-p-STAT3 (Cat#9134; Cell Signaling Technology) or rabbit IgG (Cat#2729; Cell Signaling Technology). After DNA purification, quantitative PCR was performed using primers (*Tcf7* −17.5k: 5′-CAGGGTGTTTTAGCGGGAGAG; *Tcf7* −17.5k: 5′-CAGCAACAGACACAGCCAGT; *Tcf7* + 0.2k: 5′-CAATCTGCTCATGCCCTACC; *Tcf7* + 0.2k: 5′-CTTGCTTCTGGCTGATGTCC) flanking the putative *Tcf7*-binding sites. The fold enrichment was calculated by normalizing samples of anti-p-STAT3 to normal rabbit IgG controls.

### RNA-seq

NK cells were sorted from spleen of Mst1/2^fl/fl^ (WT) and Mst1/2^fl/fl^*CD122*^*Cre*^ (cKO) mice using BD FACS Aria II (BD Bioscience). Total RNA was extracted using the RNeasy Micro Kit (Qiagen 74004) according to the manufacturer’s instructions. RNA purity and quantification were estimated using the NanoDrop 2000 spectrophotometer (Thermo Scientific, USA). RNA integrity was evaluated using the Agilent 2100 Bioanalyzer (Agilent Technologies, Santa Clara, CA, USA). Then the libraries were established using Single Cell Full-Length mRNA-Amplification Kit (Vazyme, N712-03, Nanjing, China) and TruePrep DNA Library Prep Kit V2 for Illumina (Vazyme, TD502-02, Nanjing, China) according to the manufacturer’s protocols. The transcriptome sequencing and analysis were conducted by OE Biotech Co., Ltd. (Shanghai, China). The RNAseq data were deposited in the Sequence Read Archive (SRA) repository at NCBI under the accession number PRJNA1104982.

### Statistics

The data presented above represent the mean ± SD. Two-tailed Student’s t-test and one-way ANOVA statistical analyses were performed, while Paired Student’s *t*-test was used for comparison of differences between paired samples. Statistical significance was indicated by *P < 0.05, **P < 0.01, and ***P < 0.001 respectively. All statistical analyses were performed using GraphPad Prism 8 software.

### Supplementary information

Supplementary information

## Data Availability

The RNA-seq data were deposited in the Sequence Read Archive (SRA) repository at NCBI under the accession number PRJNA1104982.
