# Supplementary material for: Hippo kinases Mst1 and Mst2 maintain NK cell homeostasis by orchestrating metabolic state and transcriptional activity
Source: Cell Death Dis. 2024 Jun 19;15(6):430. doi: 10.1038/s41419-024-06828-x (PMC11187177; doi:10.1038/s41419-024-06828-x)
Supplement: Supplementary file 1 — Supplementary information [file 41419_2024_6828_MOESM1_ESM.docx]

**Hippo** **kinases Mst1 and Mst2 maintain NK cell homeostasis by orchestrating metabolic state and transcriptional activity**

Peiran Feng^1*^, Liang Luo^2*^, Quanli Yang^3*^, Wanqing Meng^2*^, Zerong Guan^2^, Zhizhong Li^1,4^, Guodong Sun^1,4^, Zhongjun Dong^5#^ and Meixiang Yang^1,2,3,6#^

^1^Guangdong Provincial Key Laboratory of Spine and Spinal Cord Reconstruction, The Fifth Affiliated Hospital of Jinan University (Heyuan Shenhe People’s Hospital), Jinan University, Heyuan, 517000, China

^2^The Biomedical Translational Research Institute, School of Medicine, Jinan University, Guangzhou, 510632, China

^3^Guangdong Provincial Key Laboratory of Tumor Interventional Diagnosis and Treatment, Zhuhai Institute of Translational Medicine, Zhuhai People's Hospital Affiliated with Jinan University, Jinan University, Zhuhai, 519000, China

^4^Department of Orthopedics, The First Affiliated Hospital, Jinan University, Guangzhou, 510630, China

^5^The First Affiliated Hospital of Anhui Medical University and Institute for Clinical Immunology, Anhui Medical University, 230032, Anhui, China

^6^Key Laboratory of Ministry of Education for Viral Pathogenesis & Infection Prevention and Control (Jinan University), Guangzhou Key Laboratory for Germ-free animals and Microbiota Application, Institute of Laboratory Animal Science, School of Medicine, Jinan University, Guangzhou, 510632, China

Running title: Non-canonical Hippo pathway regulates NK cell homeostasis and function.

^*^These authors contributed equally to this work

^#^Correspondence to:

Dr. Zhongjun Dong, dongzj@mail.tsinghua.edu.cn.

Dr. Meixiang Yang, [mxyang@j](mailto:yangmxqilu@163.com)nu.edu.cn.

**
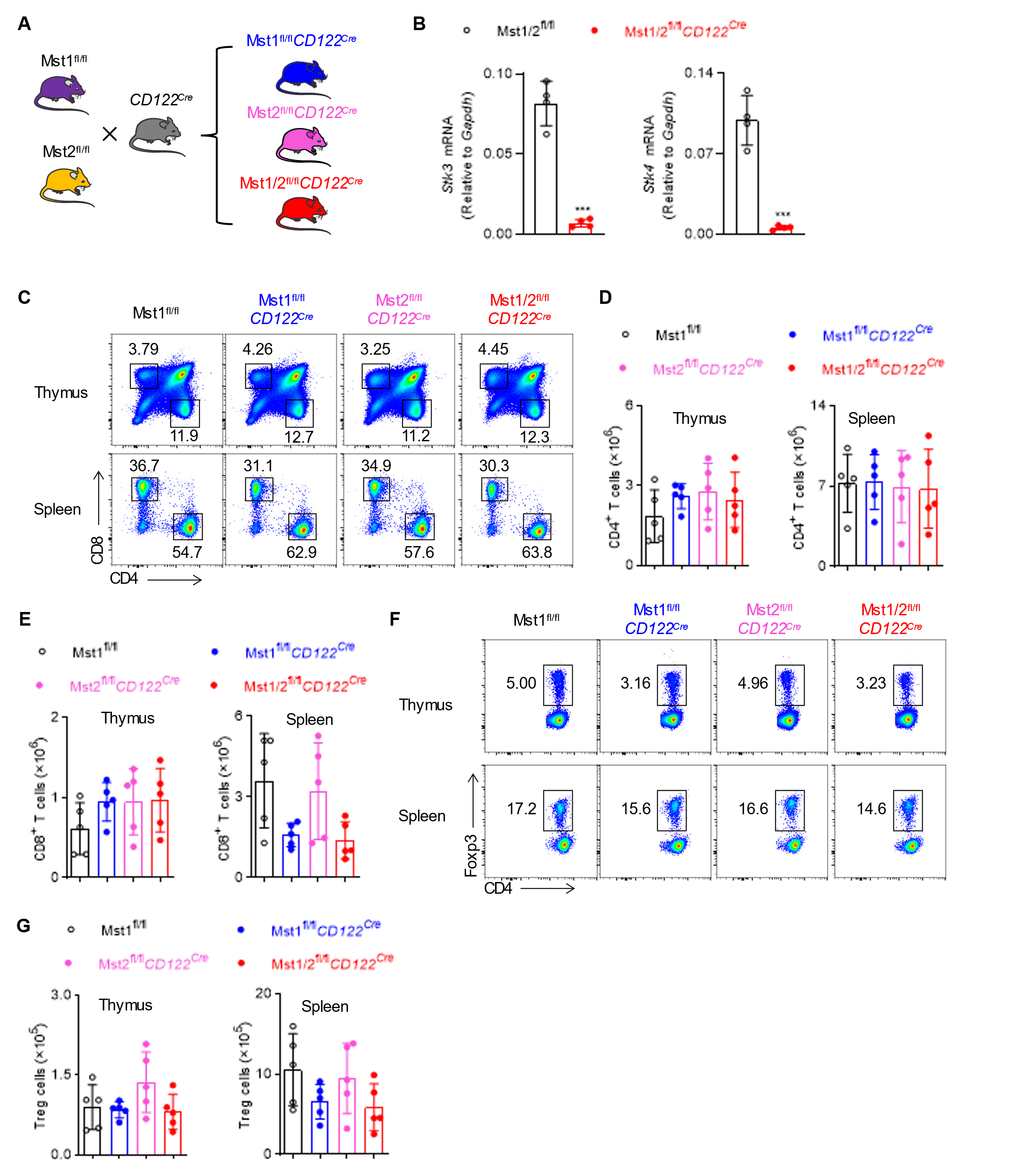
**

**Figure S1. The ablation of Mst1/2 have no impact on T cell homeostasis. (A)** Schematic graph depicts the breeding strategies for the indicated conditional knockout mice. **(B)** Quantitative PCR analysis of *Stk3* and *Stk4* mRNA expression in NK cells isolated from the spleen of Mst1/2^fl/fl^ and Mst1/2^fl/fl^*CD122^Cre^* mice (n=4). **(C-G)** Representative plots (C, F) and quantification (D, E, G) of the numbers of CD4^+^ T (D ), CD8^+^ T (E) and Treg cells (G) in the thymus and spleen from the indicated mice (n=5). Data of B-G are representative of three independent experiments with similar results.


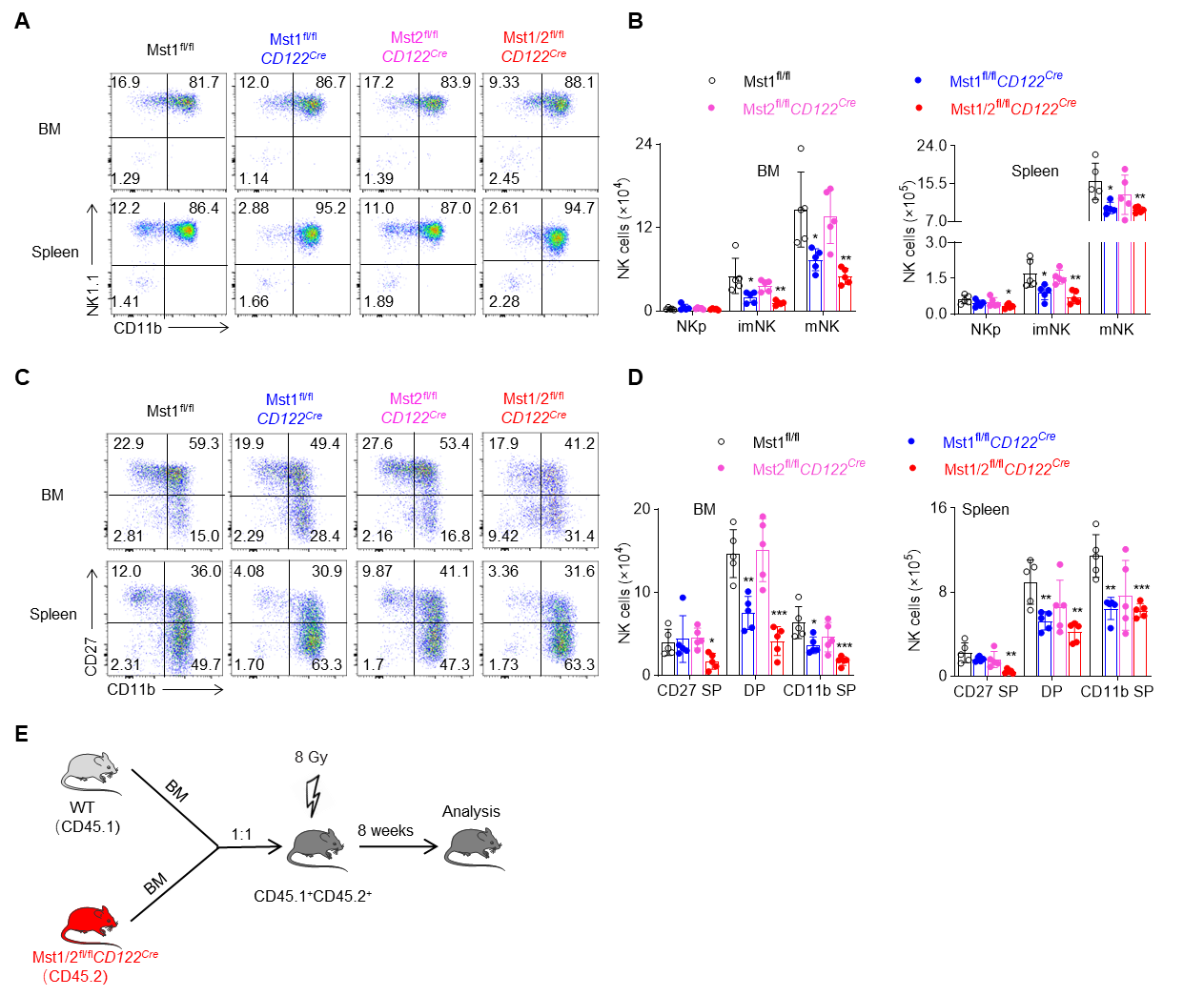


**Figure S2. Mst1/2 are crucial for NK cell differentiation. (A, B)** Representative plots (A) and the numbers (B) of NKp, imNK and mNK cells among gated CD3^−^CD122^+^ cells in BM and spleen from the indicated mice (n=5). **(C, D)** Representative plots (C) and the numbers (D) of CD27 SP, DP and CD11b SP NK cell subsets among gated CD3^−^NKp46^+^ cells in BM and spleen from the indicated mice (n=5). **(E)** Schematic graph depicts the generation of BM chimera mice. Briefly, BM cells from WT (CD45.1) and Mst1/2^fl/fl^*CD122^Cre^* (CD45.2) mice were transferred into sub-lethally irradiated CD45.1^+^CD45.2^+^ recipient mice at a 1:1 ratio. After 8 weeks, NK cells in the BM and spleen of the BM chimeric mice were analyzed by flow cytometry. Data of A-D are representative of three independent experiments with similar results.


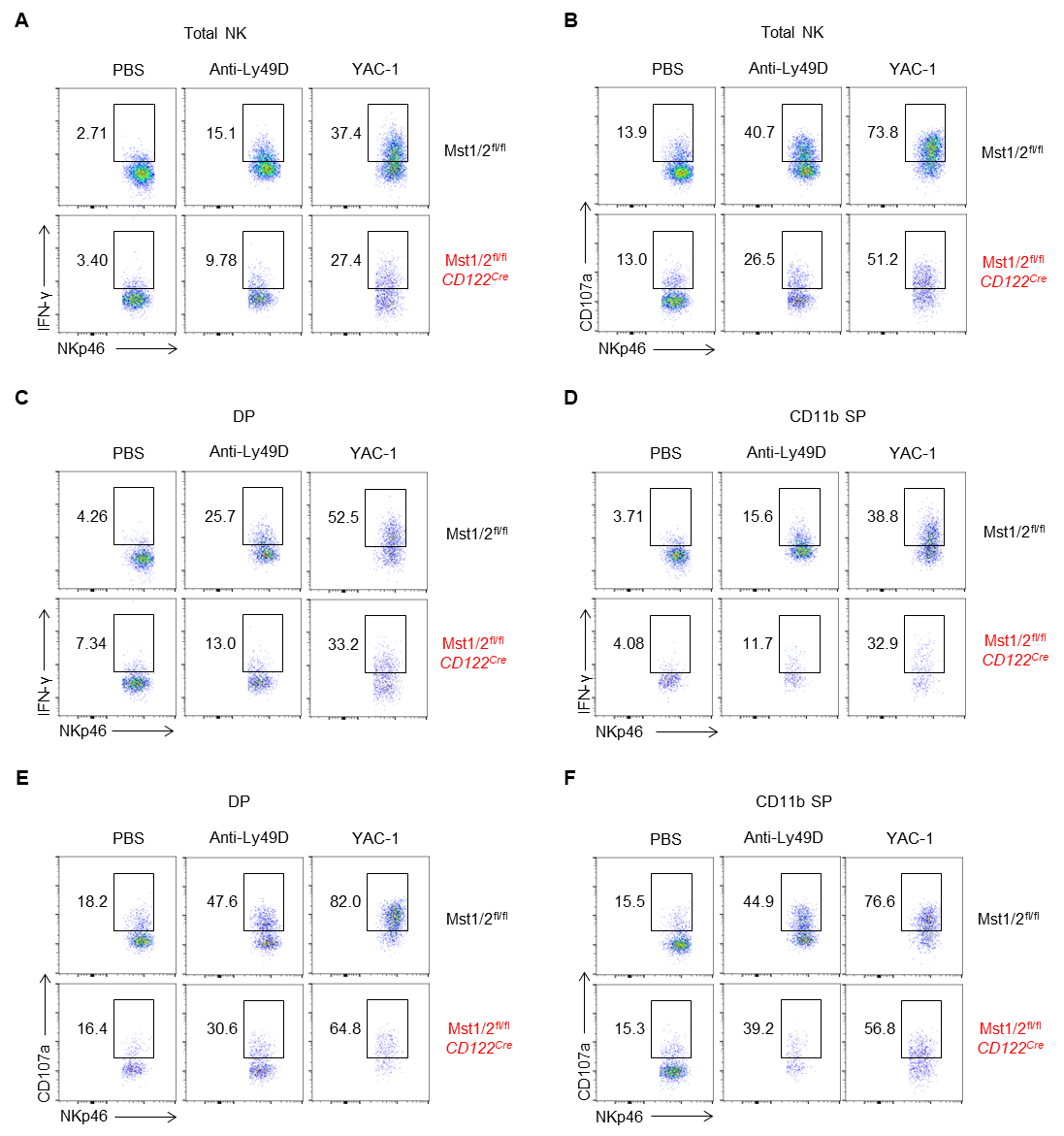


**Figure S3. Mst1/2 ablation impairs the production of IFN-γ and CD107a** **in** **NK cell. (A, B)** Representative plots show the percentages of IFN-γ^+^ (A) and CD107a^+^ (B) NK cells from Mst1/2^fl/fl^ and Mst1/2^fl/fl^*CD122^Cre^* mice. **(C, D)** Representative plots show the percentages of IFN-γ^+^ DP (C) and CD11b SP NK cells (D) from Mst1/2^fl/fl^ and Mst1/2^fl/fl^*CD122^Cre^* mice. **(E, F)** Representative plots show the percentages of CD107a^+^ DP (E) and CD11b SP NK cells (F) from Mst1/2^fl/fl^ and Mst1/2^fl/fl^*CD122^Cre^* mice. Data are representative of two independent experiments with similar results.
